# Supplementary material for: Temperature, traveling, slums, and housing drive dengue transmission in a non-endemic metropolis
Source: PLoS Negl Trop Dis. 2021 Jun 11;15(6):e0009465. doi: 10.1371/journal.pntd.0009465 (PMC8221794; doi:10.1371/journal.pntd.0009465)
Supplement: S1 Methods — (PDF) [file pntd.0009465.s001.pdf]

Supporting information: S1 Methods

Temperature, traveling, slums, and housing drive dengue transmission in a non-endemic metropolis

Juan Manuel Gurevitz*, Julián Gustavo Antman, Karina Laneri, Juan Manuel Morales

* jmgurevitz@comahue-conicet.gob.ar

Supplementary methods

Temporal variations: effects of temperature and rainfall

In order to broadly understand the temporal variations in DENV transmission, the weekly incidence rate ratio (IRR), calculated as the number of new cases during week t divided by the number of new cases during week $t-1$, was assessed in relation to temperature and rainfall. Spatial variation was not considered at this point. Temperature exerts effects on various processes involved in transmission, affecting *Ae. aegypti* development, mortality and activity, and DENV development within the mosquito (EIP). In turn, if relevant breeding sites depend on rainwater, rainfall can contribute to *Ae. aegypti* egg hatching. This will impact with a delay on transmission, since those hatched eggs need to undergo immature development to become adults, bite, become infected with DENV, go through the EIP, bite again, and transmit DENV to a susceptible person. Mordecai et al. [1] estimated the relationship between each of these processes and temperature based on numerous data gathered from the literature. This allowed us to calculate the time of hatching –and, therefore, the associated rainfall– of each mosquito cohort tentatively transmitting DENV to the observed cases. Additionally, Mordecai et al. formulated a model for the basic reproductive number, $R_0(T)$, where each term/parameter of the model is the result of the functions describing the relationship with temperature of each of these traits and of *Ae. aegypti* immature and adult mortality. We used this model to summarize the cumulative effects of temperature on DENV transmission.

For each day t we calculated the value corresponding to each temperature-dependent process according to the daily mean temperature. Immature mortality was calculated by apportioning the overall egg-to-adult survival according to the immature development rate. We considered that the case with onset of symptoms at day t got infected, on average, six days earlier, i.e., the approximate median incubation period of DENV in humans [2,3]. The *Ae. aegypti* female that transmitted DENV to that person became infected at least x days earlier (i.e., $t - 6 - x$). The value of x was the EIP duration given by adding cumulatively the daily DENV development rate. The day this sum reached one gave the EIP duration. Assuming that the *Ae. aegypti* female got infected just after becoming adult, adding the daily rate of immature development allowed estimating the approximate date of hatching and, thus, the rainfall allegedly related with transmission at day t. All the daily values of the temperature-dependent traits also enabled estimation of the temperature sensitive part of the $R_0(T)$ for each day based on the recent temperature history. We ignored the components that do not vary with temperature or time within the span of this outbreak, thus we normalized the calculated values of $R_0(T)$, rescaling it from zero to one, to provide us with a relative measure of the cumulative effects of temperature on DENV transmission.

To account for the uncertainty in the temperature-dependent traits, we used 500 random samples of the joint posterior distribution of the parameters fitted by Mordecai et al. for each trait (provided by Erin Mordecai upon request). This translated into a distribution of values of $R_0(T)$ and rainfall at hatching for each assessed week. To assess the correlation of each of these two measures with the IRR we calculated Kendall rank correlation coefficient and its significance for each of the 500 simulations. Pearson correlation was not appropriate as the assessed variables did not approximate to a normal distribution. Confidence intervals for IRR were calculated following Clopper and Pearson [4]. Confidence intervals for $R_0(T)$ resulted from the corresponding interquantile ranges of the distribution of values of $R_0(T)$ for each week.

Spatial variations: hierarchical modeling of demographic variables

The complete spatiotemporal hierarchical model considered was:

$$
\begin{aligned}
&Y_{it} \sim \text{Binomial}(y_{it}, \alpha) \\
&y_{it} \sim \text{NegBinomial}(\mu_{it}(1-\phi)\,\phi^{-1}, \phi) \\
&z_{i,t-1} = M_{i\cdot}\, y_{\cdot,t-1} \\
&\mu_{it} = y_{i,t-1} \exp\left(\beta_0 + \sum_{j=1}^{V} \beta_j v_{ji}\right) + \gamma_0 + \gamma_1 \sum_{i=1}^{N} y_{i,t-1} + \gamma_2\, z_{i,t-1} \\
&\alpha \sim \text{Beta}(2,5) \\
&\phi \sim \text{Beta}(1,1) \\
&\beta_j \sim \text{Normal}(0,1), \text{ for } j \text{ in 1 to } V \\
&\gamma_k \sim \text{Exponential}(0,1), \text{ for } k \text{ in 0 to 2}
\end{aligned}
$$

This is a hierarchical spatiotemporal regression, in which time, t, is expressed in weeks and the space unit, i, is the census fraction (see Methods in the main text for a description of this spatial unit). It is hierarchical because it considers the observation process, such that there is a latent (unobserved) variable, $y_{i,t}$, that represents the "true/real" number of cases. The observed number of cases, $Y_{i,t}$, follows a binomial distribution with detection probability α and $y_{i,t}$ number of trials. The "true" number of cases, $y_{i,t}$, follows a negative binomial distribution with mean μ_{it} and success probability ϕ, parametrizing it as the number of failures before the sequence of Bernoulli trials results in $\mu_{it}\,(1-\phi)/\phi$ successes. The negative binomial distribution is an adequate representation for possibly overdispersed random events occurring within a given timespan; additionally, it is an approximation of the binomial distribution when the binomial probability is very low (in our case because the number of inhabitants per census fraction was >100 times the weekly cases per fraction). μ_{it} is modelled as a linear function of several terms described in the main text. The number of cases in neighboring census fractions on the previous week, $z_{i,t-1}$, is obtained by the vector product between the row $M_{i\cdot}$ of the neighborhood matrix and the column vector $y_{\cdot,t-1}$. The neighborhood matrix $\mathbf{M}$ indicates with 1 when fraction i is adjacent to fraction j, such that $M_{ij} = 1$; otherwise, $M_{ij} = 0$ (including the case when $i = j$, as those cases are considered by $y_{i,t-1}$).

The model was fitted to the observed data using Markov Chain Monte Carlo sampling with Nimble package version 0.9.1 [5,6] in R version 4.0.2 [7]. Among other characteristics, Nimble allows specifying customized probability distributions. In our case, the binomial distribution of $y_{i,t}$ was customized as to deal with the situation when the model predicted $y_{i,t} < Y_{i,t}$ (i.e., "false" positives, according to the model). In such cases, the modified distribution function returned an arbitrary low

likelihood value. $y_{i,t} < Y_{i,t}$ could happen when the cases registered at cell i and week t did not all get infected in that cell, either because some did not live there –i.e., their address was misregistered– or because some lived there but got infected elsewhere, or they got infected from cases not living in fraction i or un/misregistered.

For initializing the model, the following loop for t = 1 was considered:

$$\begin{aligned} Y_{i1} &\sim \text{Binomial}(y_{i1}, \alpha) \\ y_{i1} &\sim \text{NegBinomial}(\mu_{i1}(1-\phi)\,\phi^{-1}, \phi) \\ \mu_{i1} &\sim \text{Exponential}(0, 1) \end{aligned}$$

Here, μ_{i1} comes from an exponential distribution. Initial values for Markov chains were sampled from distributions as those specified by the priors for each parameter.

Prior distributions were chosen to ensure that $\mu_{it} \geq 0$ and $0 < \alpha \leq 1$. We used weakly informative as not much could be anticipated of the effects of each term, due to mixed effects reported in the literature (see Introduction). There is abundant evidence suggesting a low but very variable probability of detecting infectious cases ranging 0.05-0.3 [8–11]. Thus, taking the estimate obtained for a world analysis of dengue incidence [11], we considered a rather wide beta distribution for parameter α with median 0.26 and with 90% of the density between 0.06 and 0.58.

The model was fitted using four Markov chains, each with 90,000 total iterations, 9,000 burn-in iterations, and a thinning interval of 3. The effective sample size and the r-hat (as indicative of convergence between chains) for each estimated parameter were calculated using the Coda package version 0.19-3 [12]. The effect of each covariate v_j was evaluated according to what fraction of the posterior samples of its β_j had the same sign as the mean of the posterior. To evaluate the adequacy of the model, we assessed whether the observed number of cases at each census fraction in each week fell within, above or below the 80% interquantile range of the predictive posterior [13] (80% provides a more stringent criterion than, for instance, 95% because 80% provides a narrower interval and, thus, a lower probability that the observed value falls within this interval). To reveal if the model captured the spatial heterogeneity present in the data, we compared the spatial distribution of these results to the spatial distribution of the weekly number of cases per census fraction. We used the mark-correlation function for this spatial analysis as it stablishes the correlation of values for a range of distances [14].

To check the robustness of the results of this model, a similar model was assessed differing only in having a Poisson instead of the negative binomial distribution. Thus, $y_{it} \sim \text{Poisson}(\mu_{it})$. This model was fitted considering confirmed and probable cases jointly, and confirmed cases only, using five Markov chains with 60,000 total iterations each. All other parameters, calculations, and analyses of the results were as described above for the model with the negative binomial distribution.

References

1. Mordecai EA, Cohen JM, Evans MV, Gudapati P, Johnson LR, Lippi CA, et al. Detecting the impact of temperature on transmission of Zika, dengue, and chikungunya using mechanistic models. PLoS Negl Trop Dis. 2017;11: e0005568. doi:10.1371/journal.pntd.0005568
2. Chan M, Johansson MA. The incubation periods of dengue viruses. Vasilakis N, editor. PLoS ONE. 2012;7: e50972. doi:10.1371/journal.pone.0050972

3. Rudolph KE, Lessler J, Moloney RM, Kmush B, Cummings DAT. Incubation periods of mosquito-borne viral infections: a systematic review. Am J Trop Med Hyg. 2014;90: 882–891. doi:10.4269/ajtmh.13-0403
4. Clopper CJ, Pearson ES. The use of confidence or fiducial limits illustrated in the case of the binomial. Biometrika. 1934;26: 404–413. doi:10.1093/biomet/26.4.404
5. Valpine P de, Turek D, Paciorek C, Anderson-Bergman C, Lang DT, Bodik R. Programming with models: writing statistical algorithms for general model structures with NIMBLE. J Comput Graph Stat. 2017;26: 403–413. doi:10.1080/10618600.2016.1172487
6. Valpine P de, Paciorek C, Turek D, Michaud N, Anderson-Bergman C, Obermeyer F, et al. NIMBLE: MCMC, Particle Filtering, and Programmable Hierarchical Modeling. 2020. doi:10.5281/zenodo.1211190
7. R Core Team. R: A Language and Environment for Statistical Computing. Vienna, Austria: R Foundation for Statistical Computing; 2020. Available: https://www.R-project.org/
8. Shepard DS, Coudeville L, Halasa YA, Zambrano B, Dayan GH. Economic impact of dengue illness in the Americas. Am J Trop Med Hyg. 2011;84: 200–207. doi:10.4269/ajtmh.2011.10-0503
9. Vong S, Goyet S, Ly S, Ngan C, Huy R, Duong V, et al. Under-recognition and reporting of dengue in Cambodia: a capture–recapture analysis of the National Dengue Surveillance System. Epidemiol Infect. 2012;140: 491–499. doi:10.1017/S0950268811001191
10. Undurraga EA, Halasa YA, Shepard DS. Use of expansion factors to estimate the burden of dengue in Southeast Asia: a systematic analysis. Halstead SB, editor. PLoS Negl Trop Dis. 2013;7: e2056. doi:10.1371/journal.pntd.0002056
11. Bhatt S, Gething PW, Brady OJ, Messina JP, Farlow AW, Moyes CL, et al. The global distribution and burden of dengue. Nature. 2013;496: 504–507. doi:10.1038/nature12060
12. Plummer M, Best N, Cowles K, Vines K. CODA: Convergence Diagnosis and Output Analysis for MCMC. R News. 2006;6: 7–11.
13. Gelman A, Carlin JB, Stern HS, Rubin DB. Bayesian Data Analysis. Chapman & Hall/CRC Boca Raton, FL, USA; 2014.
14. Wiegand T, Moloney KA. Handbook of Spatial Point-Pattern Analysis in Ecology. CRC press; 2013.
