# Supplementary material for: Temperature, traveling, slums, and housing drive dengue transmission in a non-endemic metropolis
Source: PLoS Negl Trop Dis. 2021 Jun 11;15(6):e0009465. doi: 10.1371/journal.pntd.0009465 (PMC8221794; doi:10.1371/journal.pntd.0009465)
Supplement: S1 Results — Table A in S1 Results. Hierarchical spatiotemporal modeling of confirmed cases using a negative binomial distribution during the 2016 outbreak in Buenos Aires. Table B in S1 Results. Hierarchical spatiotemporal modeling of confirmed cases using a Poisson distribution during the 2016 outbreak in Buenos Aires. Table C in S1 Results. Hierarchical spatiotemporal modeling of confirmed and probable cases using a Poisson distribution during the 2016 outbreak in Buenos Aires. Fig A in S1 Results. All confirmed and probable dengue cases during the 2016 outbreak in Buenos Aires according to origin of infection. Fig B in S1 Results. Weekly cases of dengue in Buenos Aires during the 2016 outbreak according to date of symptom onset, origin of infection and area (outside or in slums). Fig C in S1 Results. Relative incidence (cases per 100,000 inhabitants) of autochthonous dengue cases according to the value of demographic covariates and area of the city (outside or in slums). Fig D in S1 Results. Monthly number of people entering Argentina according to bordering country from October 2015 to July 2016. Fig E in S1 Results. Posterior predictive check of hierarchical spatiotemporal modeling of dengue transmission. Fig F in S1 Results. Weekly global point-pattern spatiotemporal analysis of dengue cases according to different time rings (τ) using the mark-connection function. Fig G in S1 Results. Duration of transmission foci according to the population within foci for different combinations of radii (columns) and time windows (rows). Fig H in S1 Results. Transmission foci detection under different radii and time windows (tau) in and outside slums. Fig I in S1 Results. Weekly detection of dengue transmission foci (in orange) according to local spatiotemporal analysis of autochthonous cases (black points) in Buenos Aires. (PDF) [file pntd.0009465.s002.pdf]

Supporting information: S1 Results

Temperature, traveling, slums, and housing drive dengue transmission in a non-endemic metropolis

Juan Manuel Gurevitz*, Julián Gustavo Antman, Karina Laneri, Juan Manuel Morales

* jmgurevitz@comahue-conicet.gob.ar

Supplementary results

Table A. Hierarchical spatiotemporal modeling of confirmed cases using a negative binomial distribution during the 2016 outbreak in Buenos Aires.

Variable	Mean	Q2.5%	Q97.5%	*f*	r-hat	N eff
Houses	0.43	0.25	0.66	1.00	1.01	550
No higher studies	0.30	-0.17	0.70	0.91	1.02	614
Unemployed	-0.26	-0.58	0.02	0.97	1.01	1,714
Good building qlty.	0.20	-0.18	0.67	0.83	1.01	368
Overcrowding	0.05	-0.36	0.59	0.56	1.01	307
Constant (γ_0)	0.09	0.01	0.19	1.00	1.00	2,009
City cases *(γ_1)*	0.0014	0.0010	0.0019	1.00	1.01	1,281
Neighbors *(γ_2)*	0.00045	0.00001	0.00165	1.00	1.00	16,466
Detection probability (α)	0.18	0.11	0.27	1.00	1.05	215
Prob. NegBin (ϕ)	0.45	0.30	0.64	1.00	1.01	632

The effects of the demographic covariates and of neighboring and citywide cases are displayed. The mean and 95% interquantile range of the posterior distribution of the corresponding model parameters are shown, together with the fraction *f* of the posterior with the same sign as the mean, r-hat as indicative of convergence, and 'N eff', the effective sample size.

Table B. Hierarchical spatiotemporal modeling of confirmed cases using a Poisson distribution during the 2016 outbreak in Buenos Aires.

Variable	Mean	Q2.5%	Q97.5%	*f*	r-hat	N eff
No higher studies	0.39	0.10	0.69	0.99	1.01	693
Houses	0.30	0.19	0.43	1.00	1.02	949
Unemployed	-0.16	-0.37	0.03	0.95	1.00	1,855
Good building qlty.	0.15	-0.16	0.43	0.84	1.03	401
Overcrowding	-0.03	-0.35	0.26	0.57	1.03	347
Constant (γ_0)	0.059	0.005	0.126	1.00	1.00	1,980
City cases *(γ_1)*	0.0010	0.0007	0.0013	1.00	1.01	1,397
Neighbors *(γ_2)*	0.00047	0.00001	0.00172	1.00	1.00	20,597
Detection probability (α)	0.18	0.13	0.24	1.00	1.05	321

The effects of the demographic covariates and of neighboring and citywide cases are displayed. The mean and 95% interquantile range of the posterior distribution of the corresponding model parameters are shown, together with the fraction *f* of the posterior with the same sign as the mean, r-hat as indicative of convergence, and 'N eff', the effective sample size.

Table C. Hierarchical spatiotemporal modeling of confirmed and probable cases using a Poisson distribution during the 2016 outbreak in Buenos Aires.

Variable	Mean	Q2.5%	Q97.5%	*f*	r-hat	N eff
No higher studies	0.32	0.08	0.57	0.99	1.01	710
Houses	0.25	0.15	0.36	1.00	1.01	990
Good building qlty.	0.16	-0.11	0.41	0.88	1.01	500
Unemployed	-0.07	-0.23	0.08	0.83	1.00	2,181
Overcrowding	-0.06	-0.34	0.20	0.68	1.01	408
Constant (γ_0)	0.069	0.005	0.152	1.00	1.00	1,529
City cases *(γ_1)*	0.0008	0.0006	0.0011	1.00	1.01	1,252
Neighbors *(γ_2)*	0.00054	0.00001	0.00198	1.00	1.00	18,831
Detection probability (α)	0.18	0.13	0.24	1.00	1.09	291

The effects of the demographic covariates and of neighboring and citywide cases are displayed. The mean and 95% interquantile range of the posterior distribution of the corresponding model parameters are shown, together with the fraction *f* of the posterior with the same sign as the mean, r-hat as indicative of convergence, and 'N eff', the effective sample size.

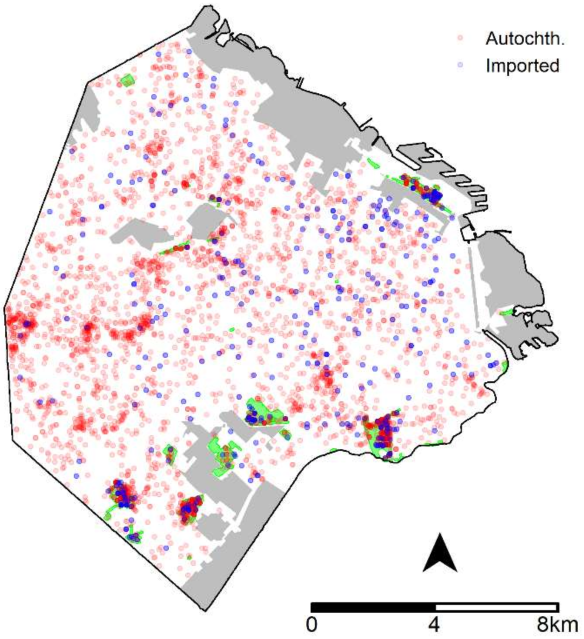

Fig A. All confirmed and probable dengue cases during the 2016 outbreak in Buenos Aires according to origin of infection. The location of each case (point) is the reported residential address. Shaded grey areas are uninhabited. Green polygons indicate slums. Map base layer downloaded from the open-data repository of the Government of the City of Buenos Aires (https://data.buenosaires.gob.ar/dataset/informacion-censal-por-radio).

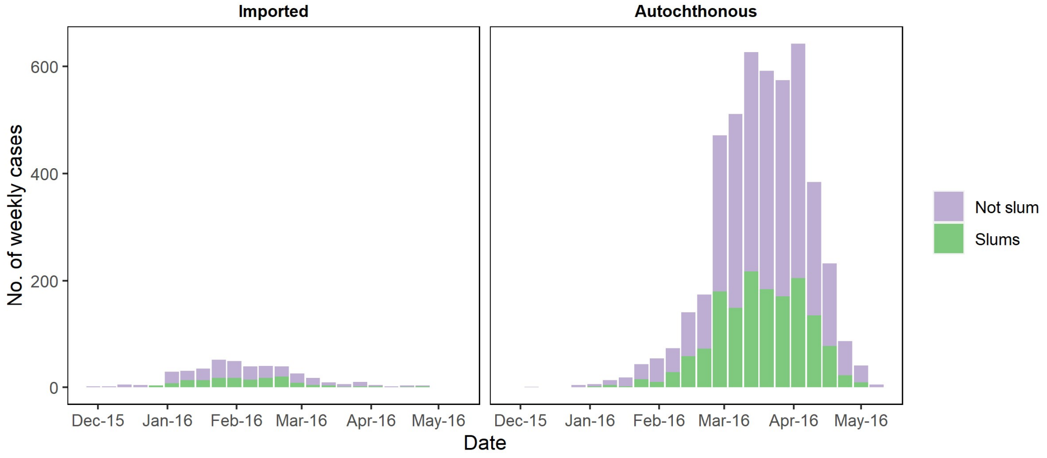

Fig B. Weekly cases of dengue in Buenos Aires during the 2016 outbreak according to date of symptom onset, origin of infection and area (outside or in slums).

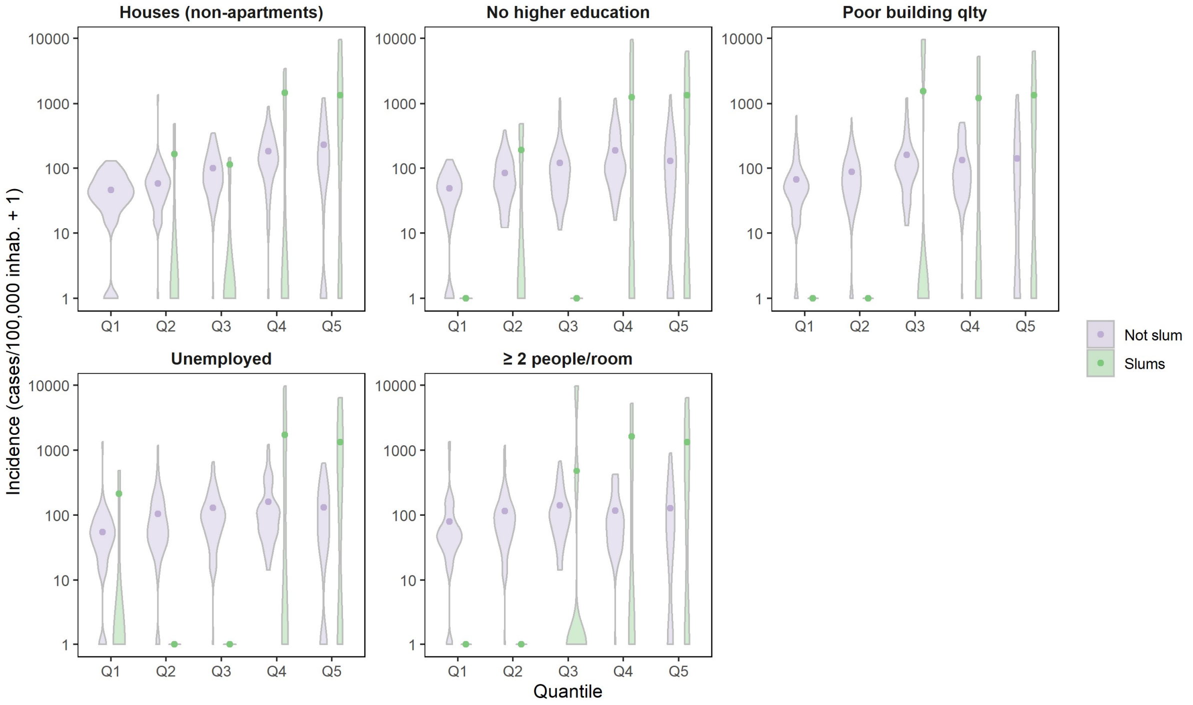

Fig C. Relative incidence (cases per 100,000 inhabitants) of autochthonous dengue cases according to the value of demographic covariates and area of the city (outside or in slums). The range of values of each covariate was divided into five quantiles. The points indicate the overall mean incidence of each quantile and area. The shaded areas denote the distribution of incidence values among census fractions. Covariates were expressed as fraction of houses (or population) with the indicated characteristic.

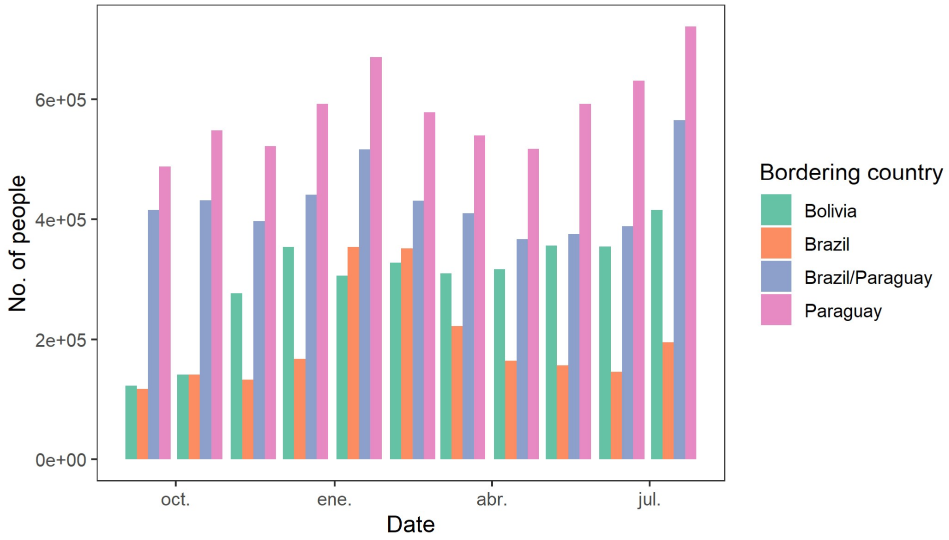

Fig D. Monthly number of people entering Argentina according to bordering country from October 2015 to July 2016. Only terrestrial checkpoints with countries with registered dengue transmission were considered. The category "Brazil/Paraguay" refers to one checkpoint that connects with Brazil but is widely used to go from and to nearby Paraguay.

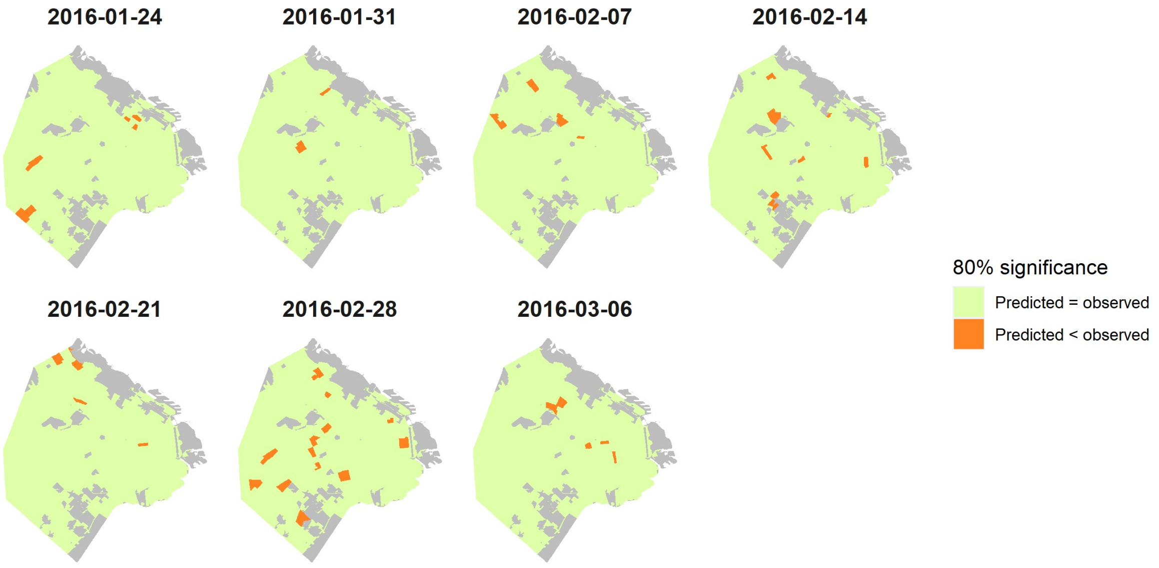

Fig E. Posterior predictive check of hierarchical spatiotemporal modeling of dengue transmission. Based on 6,480 random samples from the predictive posterior distribution, for each week (time unit) and census fraction (spatial unit) the distribution of predicted values was assessed as to whether it included the observed number of cases at 80% significance. Grey shaded areas are uninhabited or slums (not considered in the model). Map base layer downloaded from the open-data repository of the Government of the City of Buenos Aires (https://data.buenosaires.gob.ar/dataset/informacion-censal-por-radio).

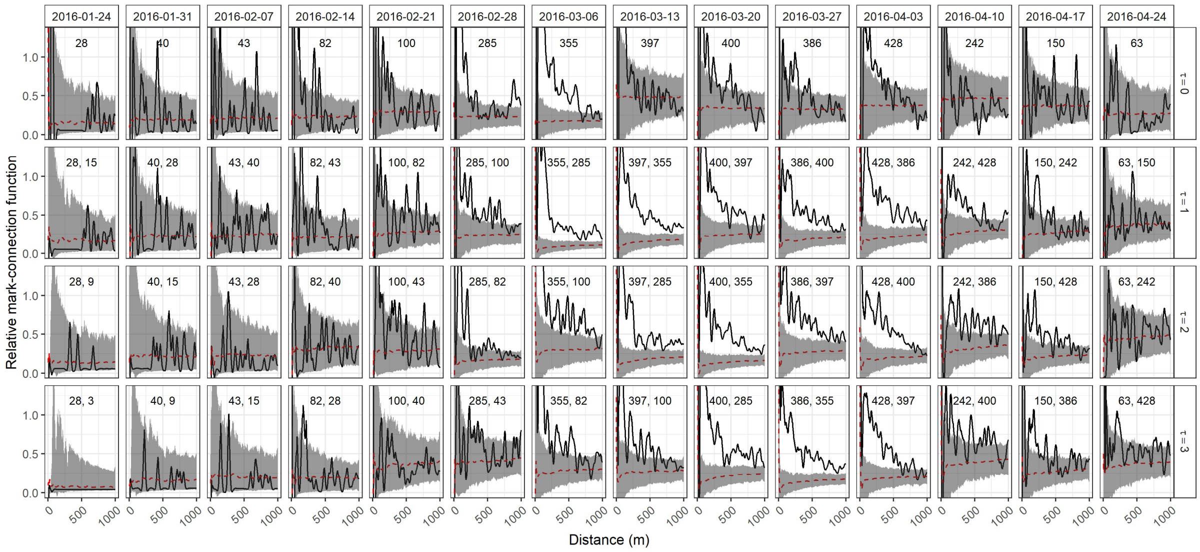

Fig F. Weekly global point-pattern spatiotemporal analysis of dengue cases according to different time rings (τ) using the mark-connection function. Only cases outside slums were considered. The black line represents the values for the observed patterns. Grey areas show the 95% confidence envelope under random labelling; the red dashed line represents the mean of the 500 random simulations. Each row corresponds to a different time ring indicated by which week was considered prior to the current week. The numbers in the plots indicate how many cases were considered in each group for the mark-connection function.

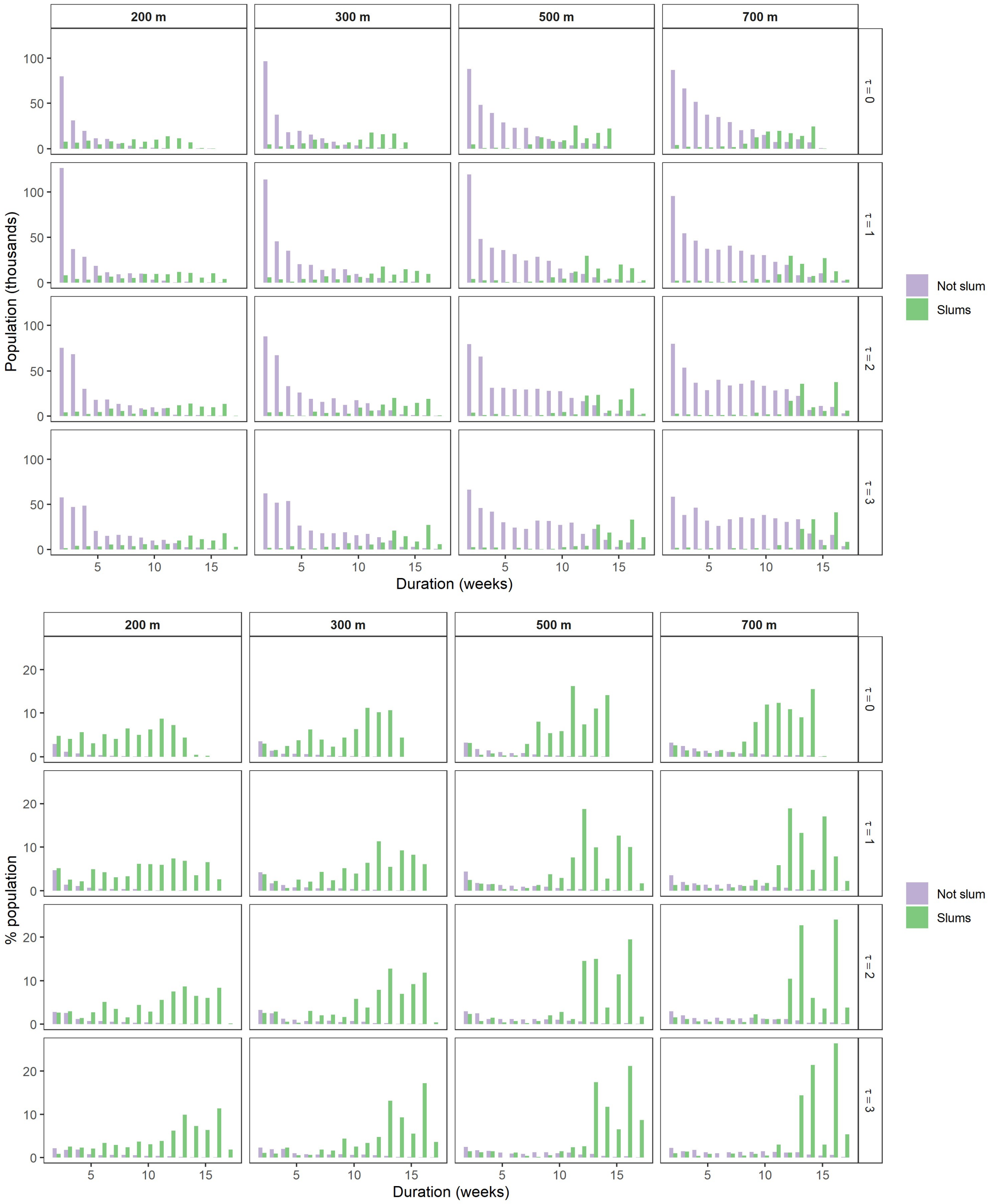

Fig G. Duration of transmission foci according to the population within foci for different combinations of radii (columns) and time windows (rows). Time window duration is indicated by τ, the number of weeks considered previous to the current. Upper panel: absolute population; lower pannel: percentage of population in each area ("Not slum" or "Slums").

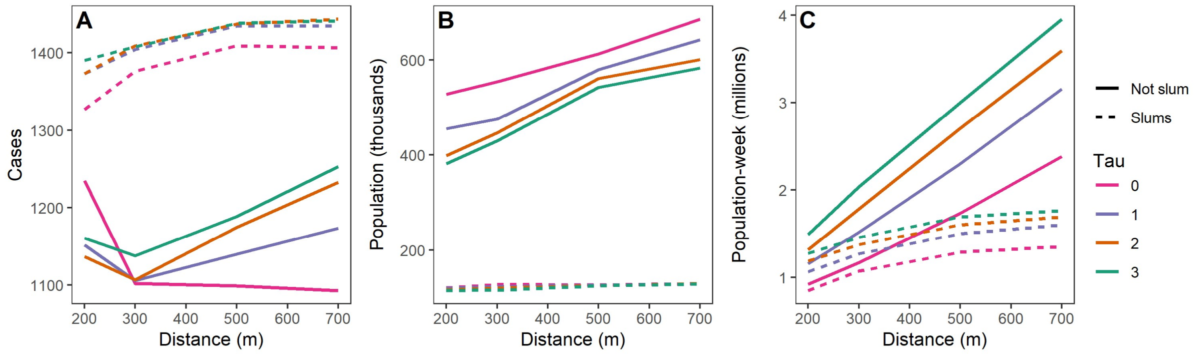

Fig H. Transmission foci detection under different radii and time windows (tau) in and outside slums. A) Total number of cases in detected foci. B) Population at least once within foci. C) Population in a focus times the number of weeks in a focus.

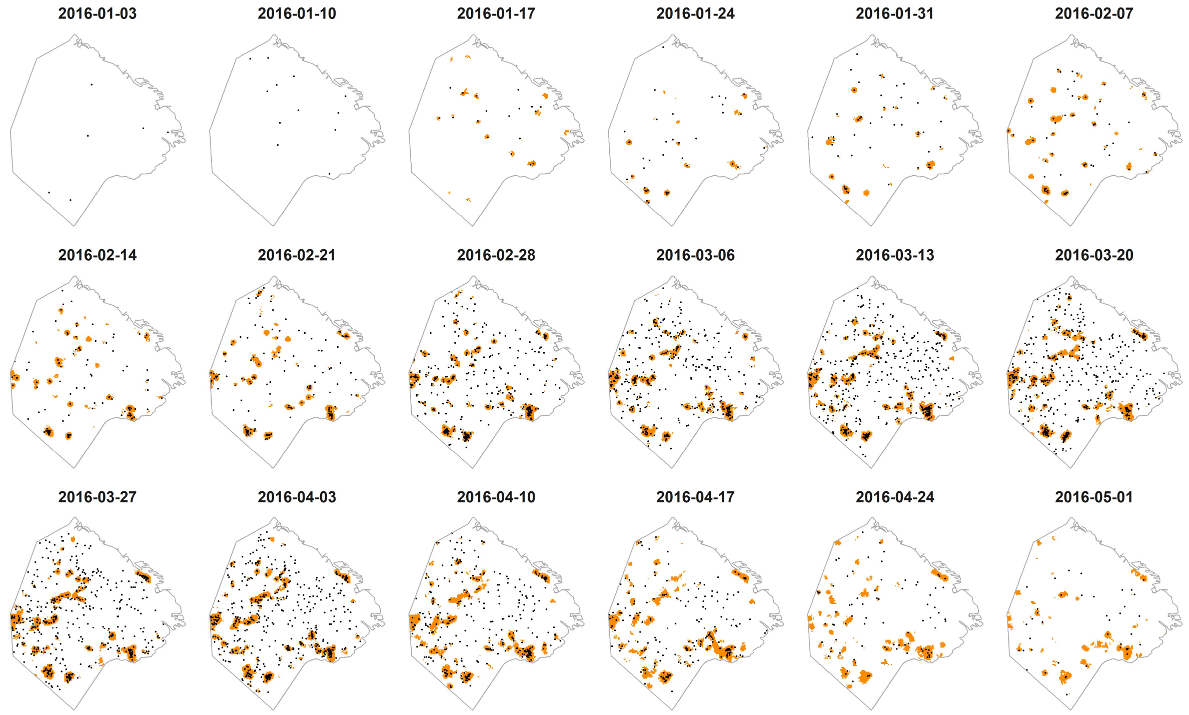

Fig I. Weekly detection of dengue transmission foci (in orange) according to local spatiotemporal analysis of autochthonous cases (black points) in Buenos Aires. The displayed dates indicate the beginning of the corresponding week. The time window considered encompassed the current week and the two previous weeks (i.e., $\tau = 2$), with a radius of 300 m. Map base layer downloaded from the open-data repository of the Government of the City of Buenos Aires (https://data.buenosaires.gob.ar/dataset/informacion-censal-por-radio).
