# Supplementary material for: Temperature, traveling, slums, and housing drive dengue transmission in a non-endemic metropolis
Source: PLoS Negl Trop Dis. 2021 Jun 11;15(6):e0009465. doi: 10.1371/journal.pntd.0009465 (PMC8221794; doi:10.1371/journal.pntd.0009465)
Supplement: Attachment — Submitted filename: Responses_rev1.pdf [file pntd.0009465.s003.pdf]

Responses to Reviewers' Comments on PNTD-D-20-01700 "Temperature, traveling, slums, and housing drive dengue transmission in a non-endemic metropolis", authored by Gurevitz JM, Antman JG, Laneri K, and Morales JM

In the following we address each of the comments of the reviewers. The original comments of reviewers are in blue italic type, whereas our responses are in black normal type. Cross-references to specific comments are indicated by the letter (reviewer #1) or number (reviewers #2 and #3) of each comment. The line numbers indicated in the responses correspond to the clear copy of the new revised version.

Methods

Reviewer #1: *This article describes the spatio-temporal pattern of DENV transmission in Buenos Aires, a large metropolis that sporadically experiences localized outbreaks. The overall design and proposed analyses are sound. However, I have some requests for clarification in the methods:*

1. Data*: I feel authors should expand more on their geocoding strategy. Specifically, they mention that cases were geocoded to the house level (I assume any address not matched to the house was discarded, as it will show as overlapping points at a 'high order' unit, such as neighborhood or Zip code).*

This brings several questions, including:

a) *Expand on how geocoding is done.*

Response: We improved this succinctly in lines 148-154: "Cases were georeferenced to their reported residential address, either street and number, intersection or slum name and block number. For cases with an address indicated by the name of a slum but with no street or block indication (14.4% of cases in slums) a random location within the slum was assigned to avoid concentrating those cases in a single point. Cases with incomplete or incongruent address (1.9% of confirmed and probable cases) could not be georeferenced and were considered in non-spatial analyses but not in spatial and spatiotemporal analyses."

b) *how were cases geocoded inside slums? One attribute of slums is that they lack proper address systems, due to lack of registry. Including such data in any geocoding platform will lead to all points being matched to the 'neighborhood' or zipcode. Unless authors can explain this in more detail, I find some of the findings (higher aggregation in slums) to be potentially driven by a bias of the geocoding algorithm used.*

Response: We appreciate the reviewer's concerns about potential bias from those cases within slums with no precise address (i.e., no block or street information). These correspond to 85.6% of the cases in slums. As mentioned in response to point a), lines 150-154 now explain this explicitly. These concerns of the reviewer made us modify slightly how we handled the 14.4% of cases in slums but with no precise location within the slum. Previously, we had placed these cases in the centroid of the corresponding slum. Now, to avoid any bias this may cause in the spatial analyses –as rightly pointed out by the reviewer–, we randomized the location of these cases (i.e., the 14.4% without precise location within the registered slum) within the area of the corresponding slum. Therefore, we updated the global spatial and spatiotemporal analyses which showed virtually no differences. The spatiotemporal hierarchical model did not show any differences as most slums lie within one single census fraction.

c) *why only confirmed cases were included? In many countries, testing stops once an outbreak is declared (or a fraction of cases is confirmed, depending on resources). I suggest running analysis with*

all probable cases to confirm that patterns observed hold. Also, if cases were confirmed by lab, I assume there are cases that were discarded due to a lab test negative. Why did authors not use both as a case-control dataset, which may have allowed for more rigorous spatial analysis.

Response: Case definitions are a matter worth mentioning and we thank the reviewer for bringing attention to it. The criteria for case definition in Argentina, at least in 2016, were different in some points to the criteria from PAHO, for instance. As we now explain in the text, "Following laboratory studies (PCR, NS1 antigen detection, and/or serology) or epidemiological assessment, cases were classified as confirmed (positive PCR or symptomatic with epidemiological link), probable (symptomatic positive to NS1 or IgM without epidemiological link), suspected (compatible symptoms without lab or epidemiological confirmation), or discarded (IgM negative or alternative diagnosis confirmed)" (lines 140-144). In fact, in the 2016 outbreak in Buenos Aires, only 411 cases were defined as probable, compared to the 4,791. Nevertheless, the reviewer' suggestion made as reconsider our criteria and, due to the very similar characteristics of confirmed and probable cases under these definitions, we now present all the results jointly for confirmed and probable cases. As can be noticed in the new version, results do not change noticeably so interpretations and conclusions remain the same.

The reviewer' suggestion regarding the use of discarded cases for a case-control design is interesting and seldom considered in the dengue literature. We anticipate some issues with our dataset on this regard. Most cases in Buenos Aires were not lab-confirmed nor lab-discarded (probably because of administrative issues to update status for those discarded). Therefore, available discarded cases are scarce (about 5% of all cases). These would hardly be informative for a spatiotemporal analysis since a single negative case (as would result on average) in a census fraction on a given week would not be reliable evidence that transmission was unlikely there at that time. We address this as a limitation in the discussion (lines 550-552).

d) *Information about the time and source of census data is required. How many years elapsed since the census and the outbreak?*

Response: Lines 159-160 indicated "Demographic data at census tract level were obtained from the 2010 national survey [49]", including reference to the online database for accessing the data. Given that the census took place on October 2010, less than six years elapsed until de 2016 outbreak. As we now explain in the text, "the demographic data used, on which the spatial analyses rely, could be outdated six years after the census. However, the low rate of population growth (<0.3% per year) [86] in Buenos Aires suggests no major biases on the results." (lines 555-557). However, population in slums in 2016 was ~30-40% larger than obtained from the 2010 census data (https://vivienda.buenosaires.gob.ar/integracion/). Therefore, we have corrected the demographic data for slums accordingly and updated the spatial and spatiotemporal analyses. This correction did not have any noticeable impact on the results.

2. Analysis. *In general, analyses are sound. Some more detail is needed to better understand their applicability. For instance:*

e) *Models appeared to run at the census tract level, but if one sees the distribution of cases it may be logical to expect that cases distribute following a zero-inflated negative binomial distribution. Please explain how zeros were handled.*

Response: As the reviewer correctly mentions, the highly spatially aggregated distribution of cases results in a high frequency of census fractions with zero cases most weeks. In our hierarchical model zeros can appear in four ways: i) unfavorable conditions of the census fraction (i.e., small beta terms, given a low contribution of neighbors and overall city cases), ii) absence of cases on the previous week, iii) the Poisson distribution, particularly, but not only, with low lambda, generates zeros, and

iv) the binomial distribution of the observation process generates certain fraction of zeros. The posterior predictive check we originally performed, together with the spatiotemporal analysis of model residuals, demonstrate that the model is handling zeros acceptably (now addressed in lines 371-375). That is, one could argue that there is no need to "inflate" the number of zeros. Furthermore, an advantage of the chosen model is that zeros can appear by different paths, each with a biological or epidemiological interpretation. Instead a zero-inflated model would state that zeros can appear randomly with no clear conceptual interpretation.

We appreciate that the Poisson distribution can be too restrictive, so we took the suggestion of using a negative binomial for modelling new cases and re-run the analysis. The new model performed well (as assessed by the posterior predictive check), and we now present this model in the results instead of our previous one. It is worth noting that results from both models are very similar. Also, considering only confirmed cases or confirmed and probable cases had no relevant impact on the results of these models (lines 374-375).

Also

Given that the main significant effects are not spatio-temporally varying, applying such an involved model (hierarchical spatio-temporal) may have given rise to similar results than a simpler spatial model with a random intercept. Perhaps I am wrong, but a justification of why spatio temporal models are applied in the presence of variables not varying ins space-time is required. Additionally, other approaches for adding spatial structure (more amenable for simulating human movement) such as correlated random walks may have shown different patterns emerge, compared to a simple 'queen' contiguity scheme. Please, justify your choice of spatial effects on your model.

Response: These are some interesting points. Certainly, the assessed covariates vary in space (among census fractions, our spatial unit) and do not vary in time. However, if cases were pooled across time (i.e., a spatial non-temporal model), it would not be possible to distinguish if a given census fraction showed no cases because it was unfavorable for transmission or because, in spite of being favorable, no DENV arrived in order to start local transmission (as explained in lines 201-207). Thus, a purely spatial model could still evidence the effects of covariates, but these would be much blurred by the high frequency of zeros. Adding a Bernoulli for zero-inflation would not help reducing this "blurring" as the Bernoulli flips the coin with the same probability for every census fraction, irrespective of its characteristics or history. As for random intercepts, we only figure that this could be random among census fractions. In this case, we would have *N* random intercepts (one for each of the *N* census fractions) plus V coefficients (one for each of the *V* covariates). Thus, we would have more parameters to fit (at least, *N*+*V* parameters) than observations (*N* observations in total, because we would have one observation per census fraction since we would have pooled over time to get a spatial non-temporal model). However, we may be missing something in our argument and we welcome any suggestions.

Considering random walks, as suggested by the reviewer, is interesting and surely allows for a richer representation of human mobility than the contiguity model we chose. At the moment, we find several issues with the implementation of random walk models for the current data: i) no available data on human mobility to parameterize the random walk model; ii) although we have no data, it is expected that people move as a random walk within short distances around given points (e.g., home, work, friends) but "jump" between this points, something very difficult to parameterized presently, and iii) an individual-based model, as needed for random walks, adds considerable complexity. Thus, given the available data we consider that our approach is a reasonable trade-off between sophistication and precision for addressing our objectives.

f) *More explanation is needed to understand the parameter alpha in the model. From the description of methods and Suppl, it is not clear to me how alpha becomes a detection probability. The model is formulated at weekly spatiotemporal slices, and there is no explanation of how such observation aggregates to provide a total alpha for the city. I assume you are averaging detectability throughout the outbreak, but please clarify how the main effect in the model translates in a detection probability. How estimates change if all cases (rather than only those geocoded) are used? is alpha scaled linearly or exponentially to the increase of N?*

Response: We appreciate these concerns. We assumed that detection of cases is not perfect, so an unknown fraction of cases remains undetected, either because of misdiagnosis, being asymptomatic or did not go to for medical attention. Our approach to deal with imperfect detection considers that, on average, only a fraction (alpha) of all cases is detected. This can be represented by a binomial distribution with probability alpha. In our model we assume that this probability is constant over time and space. Surely, this is a big assumption, as it is expected that detection varies in time (at the onset of the outbreak health practitioners may diagnose better the fewer cases, although they might be less aware of the possibility of dengue; later on the outbreak, practitioners can be overwhelmed but also more prone to diagnose dengue) and space (e.g., different social groups may have different tendency or possibilities to seek for medical attention). Our data is insufficient to model detectability in space and time. However, we considered that modeling detectability uniformly was better than not considering it at all. We now address these matters succinctly in lines 229-231: “This was a way of accounting for missing observations of cases. Thus, α would be the probability of observing/detecting a DENV case in a week given that it was present in that week”.

The detection process (represented by the binomial distribution) is part of the hierarchical spatiotemporal model. This allows considering only cases that could be georeferenced. As we now explicitly state in lines 152-154: “Cases with incomplete or incongruent address (1.9% of confirmed and probable cases) could not be georeferenced and were considered in non-spatial analyses but not in spatial and spatiotemporal analyses”. Assuming these few cases are randomly distributed in space and time and given the low proportion they represent, we expect their exclusion to have a negligible influence on the results. In any case, they would be taken into account in the detection process modeled.

g) *spatio-temporal analyses seem correct, but more detail is needed to justify the use of 'mark-connection functions' . Also, results from this function are not clearly shown (just in text, but no reference to actual values or test statistic).*

Response: In lines 254-260 we improved the justification of the mark-connection function in general: “The mark-connection function evaluates the spatial distribution of qualitatively marked points within a ring of given radius and width. This contrasts with more popular statistics (such as Ripley’s *K*- and *L*-functions) that evaluate distribution within a disc of a given radius. The disc implies that effects at shorter distances obscure effects at larger distances [59]. Considering a ring avoids this issue; thus, the mark-connection function enables characterization of patterns by assessing independently for each distance the contribution to the observed spatial pattern.”

To clarify the purpose of the spatiotemporal global analysis (with the mark-connection function), we further edited the text in lines 270-276: “To define what radius and time window to use, the average distance and time of clustering due to local transmission at a weekly base was characterized performing a global spatiotemporal analysis using the mark-connection function. The temporal dimension was incorporated by considering all cases within a given space-time ring analogous to the spatial ring. The initial phase of the outbreak would provide the best estimates of

typical clustering distance since at that time most clusters were still small, more likely reflecting the transmission pattern from a few cases."

The results of the global spatiotemporal analysis are now included as a supplementary figure (S2 Figures, Fig 6).

h) *L246. THere is mention of random labeling of infected and not infected attributes of points, but all functions used (Local K) are based on cases only, unless the authors ran a bivariate local K function. Please explain.*

Response: To clarify this we improved explanations in lines 279-281: "Analyses only evaluated the location of infected individuals (either observed or simulated). The random labeling ensured that the simulated random patterns accounted for the underlying population distribution". Thus, local *K* was always calculated for individuals labelled as infected. Comparing the local *K* of the observed pattern to the local *K* of patterns simulated by random labelling allowed assessing clustering when the observed local *K* exceeded the 95% CI of the simulated patterns.

Reviewer #2: *At the beginning of the paper, the authors defined a clear objective: to describe the main dynamic/process of dengue transmission in a non-endemic location in Argentina using a spatial, social, and climatic approach. They designed a detailed methodology that is easily followed during reading, also provides a good description of the study zone and its corresponding variables.*
The methodology proposed seems to follow a clear line from the objective to the data analysis and presented results.
Even so, the paper presents only one gap in a method description, that I think could be fixed during the revision process (that I described in Editorial and Data Presentation Modifications)

Response: We thank the reviewer for these comments. We addressed the mentioned gap as explained further below in Editorial and Data Presentation.

Reviewer #3: *(No Response)*

<u>**Results**</u>

Reviewer #1: *Analyses math what described in methods, and graphics are well designed. I just have some suggestions for improvement:*

i) *For R0, I believe there is a missed opportunity here. It would be good to find a way of determining statistically that there is an association between temperature and R0 being higher than 0. Cross correlation analyses would tell if both time series are associated, but other analyses may be more informative.*

Response: Following the reviewer' suggestion, we added a correlation analysis (methods described in S1 Methods and results presented in lines 340-341 and 348-349 of main text) which gives the missing quantitative support.

j) *As mentioned on previous section, explain the rationale of developing a hierarchical model, but more importantly describe findings in more detail. How well the model fitted the data? what is the distribution of random effects, are they spatially autocorrelated? Providing a measure of fit of the model to the data would help identify whether this is the most sound statistical approach to address the question of interest.*

Response: We acknowledge that the performance of the model could be better assessed, so we

added a spatial analysis of the residuals of the model. In S1 Methods we explain the analysis: "To evaluate the adequacy of the model, we assessed whether the observed number of cases at each census fraction in each week fell within, above or below the 80% interquantile range of the predictive posterior [13] (80% provides a more stringent criterion than, for instance, 95% because 80% provides a narrower interval and, thus, a lower probability that the observed value falls within this interval). To reveal if the model captured the spatial heterogeneity present in the data, we compared the spatial distribution of these results to the spatial distribution of the weekly number of cases per census fraction. We used the mark-correlation function for this spatial analysis as it stablishes the correlation of values for a range of distances."

In lines 372-374 from the main text we present the results of analyzing residuals: "Model residuals distributed randomly in space, evidencing that the model captured the spatial structure present in the observed number of cases (Fig 5 in S2 Figures). Additionally, 98.3% of the observed values fell within the 80% interquantile range of the distribution of the predictive posterior."

k) *spatio-temporal analyses (locak K) could be explained in more detail. Need to indicate how many cases were members of a cluster. What is the average (SD) size of clusters, duration, etc. And use such values to support claims of differences between slums and no slum areas. Also, indicate that foci are areas where locak K was significant (that was my interpretation of it, am I wrong?).*

Response: These are very good points. The main purpose of the local spatiotemporal analysis was to serve as an operational tool to identify in real-time areas with more cases than average (while accounting for population distribution) and, then, to allow prioritizing control interventions within the city (lines 261-263). Nevertheless, we now added the suggested information from local *K* as it contributes to a better picture of transmission in Buenos Aires: "Overall, 53.1% of cases belonged to clusters. Within slums, 91.3% of cases and 80.0% of the population were in clusters; outside slums, 34.9% of cases and 20.4% of the population were in clusters. Exposition to DENV transmission was also longer in slums than outside slums: 41.9% of the population in slums against 0.3% outside slums were in clusters for ≥10 weeks, whereas for 1-9 weeks 38.1% in slums and 20.2% outside slums were in clusters (Fig 7 in S2 Figures)" (lines 415-419).

We added the missing criteria for significance of local *K* and the number of simulations performed: "For each analysis 500 simulations were performed and used for building 95% confidence envelopes. In the case of the local analysis, cells with a *K*-function value above the confidence envelope for that cell was considered as having clustered cases within the radius and time window specified" (lines 285-288).

L) *one of the figures in the supplement shows violin plots, and indicate mean values. Make sure you are not plotting medians, as that is what generally is shown in such plots.*

Response: Thank you. We double-checked and in the mentioned figure (Fig 3 in S2 Figures) "The points indicate the overall mean incidence of each quantile and area".

M) *L352. this paragraph mentions results that cannot be found anywhere, and are too vague to find them informative. Where are those findings shown? You cannot report in results a value of ~300m. Have to provide actual value, and deviation around it if a measure of central tendency.*

Response: We appreciate the reviewer mentioning this important gap in our presentation of the results. In Fig 6 of S2 we now display a panel of plots showing these results in detail. We refer to these results with more detail in lines 392-399: "The global spatiotemporal analysis performed weekly for different space-time rings showed that cases outside slums were not statistically clustered until the end of February, precisely when transmission rose sharply (Fig 6 in S2 Figures). Until then, cases were clustered mostly at 250-400 m within the current week as well as with respect

to cases one week earlier; clustering was less often observed for time lags of two or three weeks. During the following weeks (March and first half of April), clustering distance increased with respect to cases in the 1-3 previous weeks (as clusters consolidated and increased in size) ranging 500-800 m, while clustering within the same week became rather negligible."

M) *a sensitivity analysis of the LocalK was performed, but the results are presented to vaguely. Need to report how many cases are added as clusters with each method, how clustering distances vary, etc, before claiming that both distances used are comparable..the supplementary figure is not enough.*
Response: We acknowledge the gap pointed out by the reviewer and we now include an additional supplementary figure (Fig 8 in S2) explicitly presenting the behavior of the results (mainly as number of cases and population in clusters) under different radii and time windows. We refer to these results in lines 419-423: "Increasing the radius and/or time window assessed translated in only slightly more cases but much more population included in clusters (Fig 8 in S2 Figures). Increasing the time window slightly increased the size and duration of clusters (Fig 8 in S2 Figures), mainly because of the "memory" that widening the time window implies."

N) *I disagree with the approach to quantify the 'cost of the intervention'. To me, this component is confusing. Given you selected which cases to geocode, the number of cases per area is a subset of what you could have. Then, unless you make sensitivity analyses around that metric (and call it something else), I do not see the value of estimating the proportion of cases in clusters to the proportion of the population measured by a census that likely occurred years before or after the outbreak. You may be under or over estimating this metrics in slums much more, as population estimates may be very variable over time. Plus, I have no idea how you arrived to the 300 m as the optimum. Again, this does not bring up any information that would strengthen the paper.*
Response: The comments of reviewers #1 (point N)) and #3 (point 8.) made us reconsider the appropriateness of our analysis of the cost-benefit of hypothetical interventions within the current paper. Evaluation of the cost-benefit of interventions is a relevant subject that needs a better elaboration and a richer analysis than what we proposed. We find that it could be better addressed in a separate paper specifically focused on this matter. Thus, we removed the whole analysis aiming, also, at improving clarity of the rest of the paper (something pointed out by reviewer #3 in his general comment).

Reviewer #2: *The authors have an extensive result section with informative tables and figures, which could improve the resolution in Fig.1 S2. For each of them presented in the main results, the paper has a complete readable explanation.*
Response: We improved the resolution of Fig 1 in S2 Figures and of several others.

Reviewer #3: *(No Response)*

<u>**Conclusions**</u>

Reviewer #1:
O) *In most part, I agree with the methods and findings. However, I have a concern that impacts some of the conclusions. The authors do not include any information about vector control actions undertaken to contain the outbreak. Without such information, and given Buenos Aires is not endemic for dengue, the authors cannot conclude that temperature drove the epidemic down. Likely,*

there is an impact of vector control (even if marginal) that may have led to an interruption of virus transmission in specific hotspot areas. Then, conclusions about temperature-driven outbreak extinction are not fully justified. The R0 model of mordecai assumes no vector control actions are conducted, which probably is not the case for this outbreak. Please, clarify.

Response: We appreciate these comments. We now mention in lines 133-134: "In 2016, although no effectiveness was measured, focal control was very scarce with little chance of having any relevant effect on transmission". This is why we consider that the interventions could only have a negligible effect on transmission. For sure, the scarce interventions and/or any other environmental or behavioral change may have contributed to the end of the outbreak. However, we consider very unlikely that such hypothetical changes would have operated almost synchronously all over the city. Nevertheless, we now further highlight the need to assess several outbreaks to verify solidly the impact of temperature on transmission in Buenos Aires (lines 467-471).

P) *Need to add a paragraph outlining limitations of the study, which there are many.*

Response: Our initial approach was to address each limitation separately, when dealing with each aspect of the results. However, we find that following the reviewer' suggestion allows a better discussion of several of the limitations, so we added a specific paragraph (lines 542-560): "The available data for this study brings about several limitations. Our data, as most data regularly collected by the health system, is passive, depending on those seeking health attention. This increases the number of unregistered cases (even of symptomatic and sub-clinical ones) [83]. Enhanced surveillance, if feasible at all in a city like Buenos Aires, would increase case detection. Also, only the residential address is registered, with no information of contact tracing or, more generally, mobility that would provide a more comprehensive understanding of where transmission takes place [35,77,84,85]. Thus, the analyses can only evaluate the hypothesis of transmission at or nearby home. Nevertheless, significant clustering of home locations of cases supports the importance of the residential address in transmission. The health system, usually focused on sick people, has a very partial account of discarded cases, something that would, otherwise, allow for a more rigorous spatial analyses using a case-control design. We lack entomological data that would also contribute to a more thorough picture of the transmission settings within the city. However, obtaining precise and extensive data on *Ae. aegypti* abundance in a 3-million inhabitant city is, at least, challenging. Lastly, the demographic data used, on which the spatial analyses rely, could be outdated six years after the census. However, the low rate of population growth (<0.3% per year) [86] in Buenos Aires suggests no major biases on the results. These limitations are common to many studies using (passive) health system data. It would prove helpful to find ways to obtain better, more detailed, and updated data to gain a more precise understanding of dengue transmission and to achieve a more effective control of transmission."

Besides this, we addressed other limitations separately:

- "High spatial and temporal resolution data on *Ae. aegypti* abundance would provide a more solid characterization of the high transmission areas, explaining how the sociodemographic characteristics link to the biophysical conditions that favor transmission" (lines 460-462).
- "A more thorough comparison, possibly a hierarchical modelling, encompassing several more outbreaks is essential to establish how temperature conditions transmission at these latitudes." (lines 469-471).
- "Further research on the joint time series of dengue cases in neighboring countries and Argentina, accounting for human mobility, would help explain how the epidemiology in neighboring countries influences and could be used to predict DENV transmission in Argentina" (lines 488-491).

- “Serotyping more cases during the outbreak could provide more solid evidence of the origin of DENV” (lines 515-517).
- The local spatiotemporal analysis “is helpful for detecting major areas of transmission but is blind to small chains of transmission, particularly in densely populated areas” (lines 537-538).

Q) *the public health relevance can be emphasized more. How is this information able to help Buenos Aires respond to the next outbreak? Control in mega-cities is challenging, and I encourage the authros to use the discussion section to expand on possible outcomes.*
Response: Thank you. We added a paragraph explicitly discussing the public health implications of our results: “Outside slums, our results provide evidence of sociodemographic characteristics associated with DENV transmission that allows stratifying risk to better target preventive and reactive interventions [87,88]. Interventions during an outbreak can be further focused in real-time by means of the local spatiotemporal analysis we implemented. This approach is additionally supported by our finding that in Buenos Aires ¬–as also reported in several other cities [19,20,76,77]– most transmission occurs at or nearby the household, although this same finding poses that almost half of cases do not get infected near their alleged home or there is a considerable mis- or under-registration. Our results underscore the importance of rapidly and effectively identifying imported cases –which are particularly likely when some neighboring countries have ongoing outbreaks– early during the outbreak and promptly blocking transmission in the vicinity of their address in the city. All these findings and tools may prove useful for other subtropical/temperate cities and for chikungunya and Zika viruses, as these are transmitted by the same vector and follow similar dynamics as DENV [88].” (lines 566-578).

Reviewer #2: *The discussion presented in the paper is in concordance to the obtained results and reviewed literature and follows a clear structure. For instance, they determine that some socio-economic variables are correlated with the propagation of dengue over the study population, e.g., migration and some precarious conditions; by implementing the Hierarchical modeling and global spatial distributions.*

Also, they suggested there were some limitations in their study like the implemented time series (only one dengue outbreak) that could cause that the rains do not present a direct correlation with dengue cases.

Finally, they suggested that this research methodology would be implemented for other infectious diseases transmitted by the Aedes aegypti, e.g., Zika and Chikungunya; for the design of public health strategies.
Response: We thank the reviewer for these comments.

Reviewer #3: *(No Response)*

Editorial and Data Presentation Modifications?
Use this section for editorial suggestions as well as relatively minor modifications of existing data that would enhance clarity. If the only modifications needed are minor and/or editorial, you may wish to recommend “Minor Revision” or “Accept”.

Reviewer #1: Minor revision.
R) *I encourage authors to cite and review the two references below, as they outline the importance of*

spatial heterogeneity for dengue stratification efforts, including the overlap between dengue and other Aedes-borne viruses: https://iris.paho.org/handle/10665.2/51652 ; https://journals.plos.org/plosntds/article?id=10.1371/journal.pntd.0006298 .
Response: We appreciate the reviewer' suggestion of this specific subject and these two references. We now address this matter in lines 566-578 as part of an added paragraph explicitly discussing the public health implications of our results.

S) *avoid overutilizing the word 'putative'.*
Response: We replaced its usage in several instances.

Reviewer #2: 1. *Instead of the robust/rigorous statistical method in lines 34 and 108, you might provide a brief mention of the statistical method implemented to make it clearer to the reader, e.g., a robust spatiotemporal regression.*
Response: Thank you. Lines 33 and 109-110 of the revised version were modified accordingly.

2. *You mentioned in lines 118-12 that some characteristics distinguish the 'slums'; in fact, in lines 190-193 you said that you excluded these areas to implement the hierarchical spatiotemporal model in Buenos Aires. I would like to know how you could determine which zone is a slum. There is an index or a specific factor to delimiter these zones? Please make the way you classify a slum and a standard neighborhood clearer in the methodology section.*
Response: This is a good point. In lines 166-169 we provide details on the definition of slums: "Demographic and geographical information on slums was obtained from the City Housing Institute (https://vivienda.buenosaires.gob.ar/integracion/). The distinctive characteristics for defining slums are that they have no urban planning, building regulations are not followed, and most ownership is irregular [51]." The city government defines slums based on those definitions. We used the classification and delimitation made by the city government. Additionally, all slums have names and clear limits that are socially acknowledged and coincide with that defined by the city government. In Buenos Aires this is very clear, maybe unlike cities elsewhere.

3. *I suggest to take care of the vocabulary used for some assertions related to the economic state of the neighborhoods (the presented in lines 118,190-193, 500, and so on). For example, the lines 190-193 might be re-written as "Slums were excluded because of their distinctive characteristics associated with overall poor health systems. Those exclusions allowed for a better understanding of the transmission factors of DENV in the city"*
Response: Thank you. In lines 210-212 we improved the text: "Slums were excluded as their specific social and environmental characteristics are distinctively associated with overall poor health. Excluding slums allowed better understanding of which factors were related to DENV transmission in the rest of the city.". The other lines mentioned were also reworded (e.g., lines 120 and 563).

Reviewer #3: *(No Response)*

<u>**Summary and General Comments**</u>

Reviewer #1: *Overall, a good article. Some suggestions for improvement, which primarily focus on simplifying methods and addressing limitations of analyses and data:*

T) *Keep in mind that the factors you identified in your model are a proxy for the process of virus*

transmission, which is dependent on mosquitoes density, susceptibility of the human population, and suitability for dengue introduction. One may find income is a proxy in some studies, or piped water in others. Population density is quite consistent as an important predictor, in your case because you are using case counts and more populous census tracts will have a higher probability of having a case. I consider many of the associations with census data to be dependent on the temporal scale of census information (how 'recent' it is) and the spatial unit of aggregation (how fine scale you are analyzing). I recommend that authors state the limitations and make sure to follow up in future research by validating their statistical findings with field information. THis brings me to the next point:

Response: We thank the reviewer for mentioning these important points.

We agree that the assessed covariates are, at best, causally linked to the biophysical conditions that make transmission possible. We intend to reflect this in our interpretations and did some modifications accordingly in the text: "High spatial and temporal resolution data on *Ae. aegypti* abundance would provide a more solid characterization of the high transmission areas, explaining how the sociodemographic characteristics link to the biophysical conditions that favor transmission" (lines 460-462).

We also agree that human population density can be related (at least, statistically) with dengue incidence. This is why we made sure to account for it in spatial and spatiotemporal point-pattern analyses by means of random labeling (as explained above). Instead, the hierarchical model (or any other of our analyses) did not include density as a covariate, at least explicitly. This model showed that a higher proportion of houses (i.e., a lower population density compared to areas with abundant apartment buildings) is associated with transmission which means that, at least in some contexts, lower population densities than average can statistically be associated with higher transmission.

As explained above (see point d)), the time elapsed since the last census was <6 years and, although Buenos Aires is a dynamic city, its population growth has been neglectable and changes in sociodemographic variables are, overall, very slow. Therefore, we consider that changes with respect to the census data employed can hardly have any relevant bias on our results.

U) *A key missing piece of data is entomology. Authors should make an effort to discuss this gap, as there is a network of ovitraps that monitor mosquito eggs on a fine spatial and temporal frequency.* ***Why are those data not included?*** *Validating that hotspots are areas with high mosquito density would have strengthened the article.*

Response: As the reviewer correctly mentions, there is (and was in 2016) a monitoring of oviposition activity of *Ae. aegypti* in the city. The network of ovitraps consists of ~200 traps evenly distributed along the city (thus, traps are separated by ~1 km on average) and the presence/absence of eggs is assessed weekly. There are several reasons why we did not use these data:

i) Qualitative (i.e., presence/absence) information of sparse ovitraps is hardly an indicative of mosquito abundance/density.

ii) Particularly in January-March most traps are positive (Fischer et al. 2016, "Long-term spatio-temporal dynamics of Aedes aegypti in temperate Argentina"). This does not contribute much to distinguish areas with different levels of transmission.

iii) Although such ovitrap monitoring represents a remarkable effort, its spatial resolution implies ~0.4 ovitraps per census fraction, the spatial unit we used for the hierarchical modeling. This would not provide solid information about mosquito abundance in each spatial unit.

iv) The ovitrap data are collected by a different research group and are not publicly available nor easy to be shared. Nevertheless, it might be worth including them in future analyses or, better, collect more appropriate entomological data.

We consider that delving into these issues in the text conspires against clarity and conciseness. However, it is worth mentioning in the text the potential and need of collecting detailed entomological information (lines 460-463).

V) *more generally, I would like authors to revisit some of hte metrics provided (alpha and cost of intervention), add more detail on the methods used and the report of results, and discuss in more detail the limitations of their study, as well as the public health relevance.*
Response: Alpha (detection probability) is addressed in point f) of these responses. The analysis of cost of intervention has been removed as explained in point n). The several limitations of the study are now better addressed (see point p)).

W) *make your data available.*
Response: As we explained in the submission form and following PLOS guidelines for restricted and third-party data, we provide an URL to download publicly available data (demographic and geographic) and contact information to ask for access to the restricted and third-party data (dengue case data and meteorological data).

Reviewer #2: *(No Response)*

Reviewer #3: *PNTD-D20–01700*
General comments:
In this paper, the authors model the spatiotemporal dynamics of a dengue epidemic in Buenos Aires, investigating the effect of weather and sociodemographic factors on dengue incidence. The paper seems to have 3 separate sub-sections: a temporal analysis of the relationship between R0(T) and relative dengue incidence, a spatial analysis investigating the effect of sociodemographic factors, and an analysis of the cost of interventions. My main comment is that these three analyses aren't currently tied together very coherently and the presentation of the methods lacks clarity, to the extent that the objective and relationship between these three different components is unclear.
Response: We appreciate these comments. To improve clarity, we added at the end of the introduction (lines 104-110) an overview of the different and complementary analyses performed that should make clearer the rationale of our approach: "It addresses i) how the outbreak began (characterizing the origin and timing of imported cases), ii) the temporal variation in transmission (assessed in terms of the effects of temperature and rain), iii) the spatial variation in transmission (statistically evaluating the relevance of different sociodemographic variables), iv) the spatial structure of transmission (characterizing the spatial scale of clustering), and v) how to identify hotspots in real-time while accounting for the heterogenous population distribution."

Also, we modified several subtitles of the method section and results to make more explicit the connection between the rationale presented in the introduction and the different results. In line with this, we joined under the same subtitle the results on imported cases and serotypes as these bring support to the analysis of imported cases. Besides this, each sub-section in the Methods section begins stating the purpose of each analysis followed by the rationale underlying the chosen tools. Additionally, motivated by this comment and other comments of reviewers (see point N) of reviewer #1), we removed the analysis of the cost of interventions as we concluded that devoting a single paper would ensure to address the subject in an appropriate way. Also, we find that removing it from the current work helps to overall clarity.

Major comments:
1. *Line 138 – could the authors provide definitions of confirmed, probably, suspected and discarded*

cases?

Response: In lines 140-144 we added the definitions and the corresponding reference: "Following laboratory studies (PCR, NS1 antigen detection, and/or serology) or epidemiological assessment, cases were classified as confirmed (positive PCR or symptomatic with epidemiological link), probable (symptomatic positive to NS1 or IgM without epidemiological link), suspected (compatible symptoms without lab or epidemiological confirmation), or discarded (IgM negative or alternative diagnosis confirmed) [48]"

2. *Line 144 – only confirmed cases were used in the analysis. Is there spatial variation in testing or healthcare usage that might affect the distribution of confirmed vs suspected cases?*

Response: According to the experience of researchers and some of the personnel of the healthcare system, it is sensible to assume that there is no relevant spatial bias in the confirmation rate of cases. In fact, the percentage of confirmed cases over confirmed and suspected cases ranged 60-70% for all neighborhoods. Nevertheless, we now jointly consider confirmed and probable cases because the way they were defined in Buenos Aires in 2016 made them quite similar.

3. *Line 146 – please specify which weather variables were used. The data were obtained from the airport's weather station. Can the authors also comment on how representative this one station is of the wider Buenos Aires metropolitan area? In particular, rainfall may be quite localised, even within a relatively small urban area.*

Response: Thanks for mentioning this gap. In lines 157-159 we specify the weather variables used and that the mentioned airport is within the city: "Data on daily mean temperature and daily rainfall were obtained from the National Meteorological Service for the weather station in Aeroparque, the local airport, which is located in the city and is representative of temperature and rainfall within the city". In fact, recordings at this station are very similar to those of another weather station located in the geographical center of the city. We did not use this station as it has some missing data.

Regarding the possible spatial heterogeneity in rainfall, it is not the case for Buenos Aires city and its surroundings. Using data of other weather stations in the vicinity of the city, we checked that rainfall was spatially quite homogeneous.

4. *The methods section could be presented more clearly. Initially, I was under the impression that the authors are using R0(T) to model variations in dengue incidence across space and time, but it seems this is not the case. Figure 3 seems to be the only place where R0(T) data are presented, as a global comparison of the time series. Even here, it's not clear to me that there's a clear correspondence between the weekly IRR and R0(T). I imagine this is because the authors only account for the weekly relative incidence, even though the effect of R0 may be lagged by several weeks.*

Response: This comment helps us improve the clarity of the text. We now indicate that this analysis is only on the temporal (i.e., not the spatial) dynamics in S1 Methods and in lines 181-184 of the main text: "In order to understand the overall temporal variations in DENV transmission, the weekly incidence rate ratio (IRR), calculated as the number of new cases during week t divided by the number of new cases during week $t-1$, was assessed in relation to temperature and rainfall, regardless of the spatial dimension". We made more explicit that our calculations of the R0 account for the time delay between hatching and adult biting: "By calculating the daily effects of temperature on these processes, we estimated the developmental history of the mosquitoes transmitting DENV on each given day. This enabled to account for the delay between hatching and adult biting and, therefore, to calculate the rainfall at the approximate time of hatching of each cohort" (lines 188-192) and "if relevant breeding sites depend on rainwater, rainfall can contribute to *Ae. aegypti* egg hatching. This will impact with a delay on transmission, since those hatched eggs

need to undergo immature development to become adults, bite, become infected with DENV, go through the EIP, bite again, and transmit DENV to a susceptible person. Mordecai et al. [1] estimated the relationship between each of these processes and temperature based on numerous data gathered from the literature. This allowed us to calculate the time of hatching –and, therefore, the associated rainfall– of each mosquito cohort tentatively transmitting DENV to the observed cases" (S1 Methods). Accounting for this delay allowed to consider the rain several weeks before, as rightly mentioned by the reviewer. We also added a statistical analysis (Kendall correlation) to evaluate the association of the IRR with R0 and rain at hatching, further explained in point i) of reviewer #1 and in S1 Methods. This analysis shows that there is a significant correlation between IRR and R0 but not between IRR and (delayed) rain for this particular outbreak (lines 340-341 and 348-349).

5. *In their spatial model, the authors assume a Poisson distribution for the dengue case count, but as far as I can see do not provide any evidence to show that this is a reasonable assumption. Dengue incidence data are often overdispersed so it would be useful to provide a stronger justification for this model choice.*

Response: There is certainly a point in this. We address it in detail in the answer to point e) of reviewer #1 and we acknowledge that a negative binomial distribution would be better suited than the Poisson distribution to deal with potentially overdispersed data. Thus, our results now present the fit of a model with a negative binomial distribution and, indeed, they reveal a relevant degree of overdispersion (Table 3). However, this change in the model did not impact on the results regarding the effects of covariates.

6. *In this model, the authors consider cases in the previous week in the same census fraction, cases in the previous week in neighbouring fractions, and cases in the previous week city-wide. But it's not clear to me whether the authors are aiming to model a biological process or statistical autocorrelation here. I would not expect cases in the previous week to be directly relevant to case incidence in the current week, because this is shorter than the extrinsic and intrinsic incubation periods. A biologically plausible model would need to account for the cumulated effect of cases, or preferably transmissibility, some weeks previously. So it seems to me that the authors are just using their model to account for correlation in their spatially stratified time series. If this is the case, then I find it much less compelling, and I would also want to see some evidence that a one week lag is sufficient to account for temporal and spatial correlation in the time series, before being convinced that the effects of other sociodemographic variables are free from confounding.*

Response: Thanks for mentioning this important point. When stating the aim of this analysis in the Methods section, we now explicitly mention that the purpose is "to assess the effects of demographic covariates on DENV transmission outside of slums while accounting for the temporal autocorrelation of cases" (lines 208-210).

In the paragraph before that, we explained the rationale of this approach (lines 201-207): "Assessing which variables are –statistically– related with the spatial distribution of autochthonous cases requires to account for the history of cases in each part of the city, even when the assessed variables may not change temporally. For instance, a very favorable area for transmission (plenty of *Ae. aegypti* and opportunities for human-mosquito contact) may show no cases just because no infected people introduced DENV". Thus, if we do not consider the temporal autocorrelation of cases, the modelled effects of the assessed covariates will be less distinctive as many observed zeros (i.e., no cases detected in a census fraction) could be due just to chance and not unfavorable values of the covariates.

What we observe at week *t* depends on what happened in the recent weeks (not only in the immediately previous one, of course). How many weeks in the past matter depends, among other

factors, on the specific time series of recent temperatures which will condition the development time of the mosquitoes. This would imply considering a variable delay in the model, thus introducing much complexity, maybe too much complexity for the available data. Alternatively, as our model does not intend to be mechanistic, we consider that it is a sound approximation (and simplification) to consider the cases only on the previous week as a way to account for the history of transmission (i.e., temporal autocorrelation) in each specific census fraction, following the rationale described above.

7. *A major assumption seems to be that cases were infected at their census tract of residence. Is there evidence to show that most infections in Buenos Aires occur in domestic settings or close to the place of residence?*
Response: Where transmission actually happens is certainly of utmost importance. We consider that, rather than an assumption, it is a conclusion of our study that most cases get infected at or near their home: "autochthonous cases were clustered in and outside slums (and overall) up to 600 m and 900 m, respectively (Fig 4D-F)" (lines 387-388) and "Altogether, the strong and persistent spatiotemporal clustering demonstrates that a major part of transmission occurs at home or in its vicinity, as noted elsewhere [19,76]" (lines 504-505). This is based on the extremely high spatial and spatiotemporal clustering evidenced globally and locally. If most cases got infected often in places other than their home or its vicinity, we find highly unlikely to observe such clustering of residential addresses. Our conclusion that most transmission occurs at or near home is consistent with multiple studies from different regions. Nevertheless, as we also mentioned in the discussion (lines 506-508) that further research should focus on the several cases that do not appear clustered. This would shed light on other relevant places for transmission.

8. *Line 253 – the authors estimate some measure of the cost of interventions, but this section is not very clear at all. What exactly is being assessed here and where do the cost data come from? The relevance of this part to the rest of the manuscript is not immediately obvious.*
Response: As mentioned for comment N) of reviewer #1, we find that this subject could be better addressed within a paper specifically focused on this. Thus, we removed the whole analysis aiming, also, at improving clarity of the rest of the paper (something pointed out by reviewer #3 in his general comment).

9. *Minor comments:*
10. *Line 80 – the authors mention the inconsistent results regarding factors associated with social and spatial clustering of dengue between different studies. But I think some of these inconsistencies are explained by the level of transmission in different settings. In non-endemic settings, more densely populated areas with less effective vector control are more likely to be affected first. In places that subsequently support endemic transmission, it also means that populations in these areas may gain higher levels of herd immunity over time, such that an inverse association between, say, socioeconomic level and dengue risk is seen, as has been observed in some urban settings in Brazil.*
Response: We appreciate this comment and agree that it can be quite complex to understand why social factors show different statistical associations from one place to another. Certainly, the dynamics of immunity may play a role. Additionally, as also pointed out in point T) by reviewer #1, social factors are proxies of the biophysical processes directly linked to transmission. In lines 460-462 we address this: "High spatial and temporal resolution data on *Ae. aegypti* abundance would provide a more solid characterization of the high transmission areas, explaining how the sociodemographic characteristics link to the biophysical conditions that favor transmission". Also, as part of the introduction, we briefly refer to the role of immunity as part of the intrinsic dynamics of

host-parasite interaction: “seasonality is considered to be driven by a combination of the effects of rain, which increases the availability of artificial breeding sites where immature *Ae. aegypti* develop, temperature, and the intrinsic dynamics of host-parasite interaction” (lines 65-68).
